# A cognitive account of belief: a tentative road map

**DOI:** 10.3389/fpsyg.2014.01588

**Published:** 2015-02-13

**Authors:** Michael H. Connors, Peter W. Halligan

**Affiliations:** ^1^ARC Centre of Excellence in Cognition and its DisordersSydney, NSW, Australia; ^2^Department of Cognitive Science, Macquarie UniversitySydney, NSW Australia; ^3^Dementia Collaborative Research Centre, School of Psychiatry, University of New South WalesSydney, NSW, Australia; ^4^Sydney Medical School, University of SydneySydney, NSW, Australia; ^5^School of Psychology, Cardiff UniversityCardiff, UK

**Keywords:** belief, belief formation, cognitive neuropsychiatry, delusion, schema

## Abstract

Over the past decades, delusions have become the subject of growing and productive research spanning clinical and cognitive neurosciences. Despite this, the nature of belief, which underpins the construct of delusions, has received little formal investigation. No account of delusions, however, would be complete without a cognitive level analysis of belief *per se.* One reason for this neglect is the assumption that, unlike more established and accessible modular psychological process (e.g., vision, audition, face-recognition, language-processing, and motor-control systems), beliefs comprise more distributed and therefore less accessible central cognitive processes. In this paper, we suggest some defining characteristics and functions of beliefs. Working back from cognitive accounts of delusions, we consider potential candidate cognitive processes that may be involved in normal belief formation. Finally, we advance a multistage account of the belief process that could provide the basis for a more comprehensive model of belief.

“Nothing appears more remote from the current frontiers of neuroscience than the circuits underlying the fixation and mutation of human beliefs”

(Bisiach et al., 1991, p. 1029).

## INTRODUCTION

Delusions, considered as false *beliefs,* have been the subject of study from a wide range of scientific and medical disciplines, including psychiatry, psychology, and cognitive neuroscience. While each of these approaches provides an important perspective on delusions, the nature of belief that underpins the construct remains largely unspecified. Understanding the nature of belief is of particular significance when trying to explain how delusions form. Over the past 40 years, several competing accounts have been proposed to explain delusions (Maher, 1974; Fear et al., 1996; Corlett et al., 2010; Coltheart et al., 2011). Although these highlight possible impairments – including both excesses and deficits – responsible for delusions, their primary focus has been to identify specific neuropsychological abnormalities responsible for delusions and do not explicitly address the nature of belief. As such, these accounts are necessarily incomplete as they do not consider the basis and broader influences of non-pathological belief formation. A comprehensive account of delusion will likely require reference to the processes involved in non-pathological belief formation to fully characterise the nature of the pathology.

This need for a conceptual framework to explain non-pathological belief has been previously highlighted by cognitive neuropsychiatry (Halligan and David, 2001), which locates explanations of abnormal processes (psychopathology) within a modern understanding of normal psychology. As Marshall and Halligan (1996, p. 9) wrote:

*“…normal informational processing systems are the domain over which any disorder of psychological function must be defined. The elucidation of a disorder of reasoning presupposes an account of how normal reasoning takes place...The (correct) description of failures of reality testing presupposes a theory of normal reality testing required for normal belief formation.*”

In the case of belief, an account of normal belief formation provides a framework to better appreciate delusions in a principled and testable manner. It would also, of course, need to be revised in the light of further clinical findings. Such an account, however, begs the question as to what cognitive processes might be involved in normal belief formation, how they relate to the current tasks used to measure deficits in delusion research, and the evolutionary purpose of belief. None of these provide for simple answers when trying to provide a comprehensive theory of belief and its pathologies. Indeed, it should be noted that some theorists question whether all delusions can be understood in terms of beliefs and suggest that some delusions may be better considered to be experiences, rather than beliefs *per se* (Jaspers, 1963; Parnas, 2004; Cermolacce et al., 2010; however, see also Bayne and Pacherie, 2005; Bortolotti, 2009, 2013; Langdon and Connaughton, 2013). In this paper, we discuss some of the issues involved in studying belief and provide a tentative road map of the stages of complexity that a more complete account of belief will likely need to address.

## DEFINING BELIEF

Belief can be defined as the mental acceptance or conviction in the truth or actuality of some idea (Schwitzgebel, 2010). According to many analytic philosophers, a belief is a “propositional attitude”: as a proposition, it has a specific meaning that can be expressed in the form of a sentence; as an attitude, it involves a mental stance on the validity of the proposition (Schwitzgebel, 2010). Beliefs thus involve at least two properties: (i) representational content and (ii) assumed veracity (Stephens and Graham, 2004). It is important to note, however, that beliefs need not be conscious or linguistically articulated. It is likely that the majority of beliefs remain unconscious or outside of immediate awareness, and are of relatively mundane content: for example, that one’s senses reveal an environment that is physically real, that one has ongoing relationships with other people, and that one’s actions in the present can bring about outcomes in the future. Beliefs thus typically describe *enduring, unquestioned ontological representations of the world* and comprise *primary convictions about events, causes, agency*,* and objects that subjects use and accept as veridical*.

Although obvious, beliefs are significant because they are held by us to be true and provide the basis for us to understand the world and act within it (Halligan, 2006). Beliefs, or perhaps more realistically belief systems, provide the ‘mental scaffolding’ for appraising the environment, explaining new observations, and constructing a shared meaning of the world (Halligan, 2007). Consider, for example, the fundamental and widespread effects of the transition from Ptolemaic astronomy to Copernican astronomy, from Newtonian physics to Einsteinian physics, or from a miasmatic theory to a germ theory of disease (see Kronemyer and Bystritsky, 2014). In a more immediate sense, beliefs allow us to interpret and appraise our ongoing experience, and to place our experience within a wider meaningful context involving the past and future. As such, beliefs can have significant emotional consequences. Beliefs also provide a basis for action by providing both a representation of the environment and a framework of goals and actions (Tullett et al., 2013). Given this overarching influence of belief on our experience, beliefs that are considered dysfunctional or inaccurate are often the target of psychological interventions (Beck, 1976; Young et al., 2003; Hofmann et al., 2012; Kronemyer and Bystritsky, 2014).

In everyday life, our understanding of belief is provided by a framework of folk psychology. This folk account frequently refers, in particular, to a notion of belief in understanding the thoughts and intentions of others. In community surveys, members of the general population typically endorse a relatively coherent set of belief characteristics (Pechey and Halligan, 2012b). The vast majority of subjects, for example, when asked to identify the characteristics of belief, consider it to involve a *strongly held conviction* that is *resistant to change*, provides *a veridical framework for explaining how things are or should be,* and is* capable of influencing thoughts, behavior, feelings, attitudes, and decisions* (Pechey and Halligan, 2012b). The high degree of consistency in defining beliefs in the general community is both reassuring and informative. It also supports the need for belief or a belief-like construct when accounting for how we interact with the world and each other.

Beliefs can be distinguished from other types of cognitive “representations” that are more frequently referred to in contemporary cognitive science, such as memory, knowledge, and attitudes. In contrast to memory, beliefs can apply to present and future events, as well as the past. In some cases, it may also be possible to distinguish between memories that are believed (as in the vast majority of memories) and memories that are not believed (as in false memories when a person recognises that the remembered event could not have occurred; Loftus, 2003). In contrast to knowledge, beliefs are, by definition, held with conviction and regarded as true (Fishbein and Ajzen, 1975; Eagly and Chaiken, 1993; Wyer and Albarracín, 2005). Beliefs also typically involve a large self-referential element that may not be present in knowledge. Finally, in contrast to attitudes (as understood in social psychology, rather than the broader philosophical usage), beliefs need not contain an evaluative component, which is a defining characteristic of attitudes in social psychology (Eagly and Chaiken, 1993). On the other hand, beliefs may provide a framework for understanding attitudes (e.g., the belief that an object has a particular property and the belief that this property should be evaluated in a particular way; for further discussion, see Kruglanski and Stroebe, 2005; Wyer and Albarracín, 2005). In all three cases, however, there is likely to be considerable overlap with belief and the different constructs may involve shared underpinnings. Semantic memory, for example, which involves memory for meaning, is likely to have many commonalities with belief.

## NEGLECT OF BELIEF

Unlike other cognitive processes – such as perception, memory, attention, language, and actions systems – beliefs have not received widespread empirical consideration and there is no complete cognitive account of belief (Bell et al., 2006a). There are several reasons for this neglect. The first may stem from the philosophical debates around the nature of belief itself (Churchland and Churchland, 2013). There is, for example, no philosophical consensus on what belief is (McKay and Dennett, 2009) or even what constitutes a delusion (Spitzer, 1990; David, 1999; Coltheart, 2007). Whereas some philosophers have argued that our folk psychological understanding of belief is more or less accurate (Fodor, 1975; Dretske, 1988), others have argued that it is wrong and will be superseded by a radically different theory with the advancement of neuroscience (see Churchland, 1981; Baker, 1987, 1995; Churchland, 1999; Dennett, 1999; for a discussion of these issues, see Bell et al., 2006a; Schwitzgebel, 2010). It is important to note, however, that most of these accounts do not deny that the scientific investigation of belief is possible (see, however, Stich, 1983). Instead, the accounts offer different predictions about what future scientific investigation will uncover as the basis of what we call “belief” and how this will relate to current common-sense understanding. Even the “eliminativist view,” which holds that the ‘folk’ understanding of beliefs is mistaken, predicts that our ‘folk’ understanding of belief will be replaced by a better specified neuropsychological theory.

Another reason for the neglect of belief stems from the challenges of articulating a cognitive account for a complex process that is likely to be supported by a number of component processes (Bisiach et al., 1991; Langdon and Connaughton, 2013). According to the influential views put forward by American philosopher and cognitive scientist Fodor (1983), beliefs are less tractable for study than the low level peripheral cognitive processes or modules (such as attention, memory, perception, and language). According to Fodor, “unencapsulated, central processes” such as fixed beliefs do not share the same characteristics or properties of modularity and instead draw on information from many sources (Fodor, 1983). As a result, it is difficult to elucidate the specific high level cognitive systems involved. In a similar way, Quine and Ullian (1970) proposed that beliefs are unlikely to exist in isolation and typically form an interconnected web in which beliefs “cohere” with one another to avoid cognitive dissonance. This complexity of beliefs poses challenges for empirical investigation (Damasio, 2000; Corlett et al., 2010). In practical terms, it makes it difficult to isolate beliefs from other cognitive processes and operationalise their investigation. Perhaps as a result, and despite their considerable importance for a complete description of a cognitive neuroscience, the cognitive nature of beliefs has attracted little formal investigation (Bell et al., 2006a; Brugger and Mohr, 2008; Bell and Halligan, 2013).

## A FUNCTIONAL PERSPECTIVE ON BELIEF

Despite this neglect, it is possible to identify four key, albeit overlapping functions of belief. First and foremost, beliefs provide a **consistent and coherent representation of a subject’s world** and the subject’s place within it. Such an intuitively coherent and ever-present framework allows subjects to pursue goals, avoid threats, and regulate their behavior in response to changes in their environment. This framework is presupposed by other higher-order cognitive functions, such as planning and decision-making, which require beliefs to conceptualise and evaluate the current situation, actions, and consequences. This framework thus provides the basis of action (Tullett et al., 2011, 2013). As Tullett et al. (2013, p. 401) note:

“Every action that we take is grounded in an elaborate web of beliefs and goals. Take the simple act of opening a door. Such an act depends on our beliefs about what lies beyond the door, as well as what is available to us in our current location. At an even more basic level, our attempt to open the door is rooted in a belief that we understand how a door works, and are capable of using it. Furthermore, without the goal of pursuing something beyond the door, the act of opening the door would probably not take place.”

While such a framework may often be assumed, securing a sense of meaning appears particularly critical when defining one’s identity and coping with uncertainty (Inzlicht et al., 2011).

Second, as a stable representation, beliefs provide an **explanatory framework** for interpreting the world and processing incoming information. When faced with situations that threaten the coherence of the collective framework, subjects typically attempt to resolve inconsistencies by seeking to restore the over-arching sense of meaning. The coherence provided by the subject’s web of beliefs allows the subject to quickly integrate and, if necessary, reconcile new observations with previous observations held in memory. In this way, collective representations can evolve over time in response to new experiences, yet still represent the subject’s pooled understanding based on the past. This adaptive function allows subject’s greater capacity to understand and adjust to their environment. It also allows a subject to quickly interpret ambiguous or incomplete information and respond accordingly. Beliefs thus allow subjects to go beyond the available sensory information and act effectively in their environment.

Third, at a more basic level, the explanatory framework of beliefs helps to **configure and calibrate lower-level modular cognitive systems**, such as perception, language, memory, and attention. Beliefs provide the interpretive “lens” that shape our experience of the world. Consequently, beliefs are not just the reportable end-product of cognitive processes; they also generate expectations that help define on-line sensory experience through top–down processing. It is well established that phenomenological experience is not simply the registration of sensory inputs through domain specific transducers, but rather the constructive integration of sensory information filtered through pre-existing beliefs. This is nicely illustrated in visual illusions: a large body of research has shown that perception of an object or scene is not determined solely by the empirical sensory information, but rather is subject to top–down processes and expectations (Gregory, 1997). In the same way, our beliefs about the world prefigure our perceptual system. Our perception of the world thus involves the reconstruction of both sensory and pre-existing information about the world. This interpretative filter provides for the meaning, structure, and unity of immediate experience (Gregory, 1997).

Finally, at an interpersonal level, beliefs serve important **social functions**. In addition to allowing subjects to navigate social relationships and interpret other people’s motivations, beliefs provide a sense of community and security. Shared beliefs help define group norms and values. They provide a common understanding that enables interaction and facilitates social governance. They also help co-ordinate groups of individuals and provide for the development and transmission of cultural representations (see Sperber, 1997). These social functions may be particularly important in the acquisition of knowledge: they allow individuals within the community to acquire knowledge about their environment without necessarily learning this knowledge first hand and being exposed to any accompanying risks. The social functions of beliefs also means that beliefs cannot simply be understood by studying individuals in isolation and instead need to be related to their broader social context, including other beliefs in their milieu.

## CHARACTERISTICS AND DIMENSIONS OF BELIEF

Beliefs are best considered as being multidimensional. Beliefs share a number of common properties but can vary across dimensions within these properties. These include the following:

(1) Beliefs have **different origins**. Beliefs, for example, can be formed through direct experience or by accepting information from a trusted or authoritative source (Hughes and Sims, 1997; Langdon, 2013).
(2) Beliefs **vary in terms of the level of evidence** and support they command. Some beliefs have high levels of evidence, while others appear to be accepted without requiring much evidential support (Lamont, 2007).
(3) Beliefs can said to be “**held” at different levels of awareness**. Whereas some beliefs may involve considerable conscious preoccupation and rumination (susceptible to reflective control), other beliefs may appear implicit, unconscious, and only evident by inference from behavior (not susceptible to reflective control; Young et al., 2003).
(4) Beliefs **vary considerably in generality and scope**. Beliefs may refer, for example, to specific objects or individuals, groups of objects and people, or whole classes of objects and people (Freeman, 2007).
(5) Beliefs **vary in their degree of personal reference**. A belief can be limited to the specific individual holding the belief (e.g., “I am unique”); extend to friends, relatives and other in-group members; or apply to other groups of people or all people equally (Freeman, 2007).
(6) Beliefs can be **held with different levels of conviction or degrees of confidence.** This can range from firmly held (e.g., in the case of basic physical laws) to relative uncertainty (e.g., in the case of unfamiliar topics; Peters et al., 2004). In some beliefs, this conviction may even fluctuate over time or across different contexts (Bisiach et al., 1991; Connors and Coltheart, 2011).
(7) Beliefs **vary in their resistance to change** in response to counter-evidence and social pressure. While related to conviction, people can also vary in how open they are to disconfirming evidence toward their belief and to considering alternative points of view.
(8) Beliefs can **vary in their impact on cognition and behavior.** This may likewise be influenced by degree of conviction. Whereas people may act on some beliefs, they may fail to act on other beliefs that they verbally endorse (Bortolotti, 2013).
(9) Beliefs can **produce different emotional consequences.** Whereas some beliefs may be relatively innocuous or even self-serving, other beliefs may cause considerable distress (Beck, 1976).
(10) Beliefs vary in the degree to which **they are shared by other people**. Whereas some beliefs are very common, other beliefs may be comparatively unusual (e.g., in the case of some delusions; David, 1999).

It remains to be seen how these different properties are cognitively and neutrally instantiated. It is possible, for example, that some properties reflect qualitatively distinct subtypes of beliefs. It is also possible that some properties instead simply reflect variation along a continuum within a single type of belief.

A particularly important feature of beliefs is their consistency and interrelationship with one another. According to Quine and Ullian (1970), beliefs form an interconnected web in which beliefs somehow “cohere” with one another to avoid discord. This is supported to some extent by empirical evidence, which indicates that the degree of co-endorsement of beliefs within thematic groupings is greater than random occurrence (Pechey and Halligan, 2012a). In a similar way, Thagard (2000) has argued that beliefs cohere with other beliefs that jointly support each other and extends the notion of consistency to a wider range of cognitions, including those involved in perception and decision-making. The acceptance or rejection of beliefs thus depends on maximizing their coherence with both beliefs and other representations. A related notion of consistency is also present in Festinger’s (1962) cognitive dissonance theory, which suggest that humans are strongly predisposed to seek consistency among their beliefs, particularly when holding contradictory beliefs might compromise self-esteem (Cooper, 2007).

The degree of coherence between beliefs also has implications for interpreting and studying individual beliefs in isolation. A particular belief, for example, may entail a number of similar beliefs on related topics. Indeed, some philosophers have argued that beliefs can only be understood by relating them to a background of other beliefs and desires (referred to here as a holistic account; Davidson, 1973, 1984). In this way, beliefs form part of a wider network of beliefs that restricts what new beliefs are possible (e.g., Quine and Ullian, 1970; Davidson, 1973, 1984). Other philosophers, in contrast, have argued that beliefs exist as discrete entities that are largely independent of one another (referred to here as an atomistic account; Price, 1934, 1969). In this way, a person may hold seemingly contradictory beliefs. While there is empirical evidence of some degree of coherence of belief, the extent to which beliefs are dependent on each other remains an important theoretical question (Pechey and Halligan, 2012a). It also has important implications for research: Whereas a holistic account, for example, suggests that a particular belief will involve widely dispersed neural activation, an atomistic account suggests that the relevant neural activation will be relatively circumscribed (Bell et al., 2006a).

Another significant issue for studying the properties of belief is the degree to which subjects are aware of their beliefs. In pragmatic terms, a person’s beliefs are often taken to be what they themselves declare them to be. This type of explicit expression, however, requires insight, reflection, and memory of the belief, as well as linguistic representation. The vast majority of beliefs, however, are not likely to be conscious or reportable, but instead simply taken as granted without reflection or awareness. Such beliefs may be inferred from a subject’s behavior, but otherwise remain unconscious and enacted largely involuntary. This automaticity also applies to the formation of new beliefs. We cannot, for example, choose our beliefs – we cannot choose to believe that it is raining if it is not – and instead often discover our beliefs when we reflect and consider what they are (see Engel, 2002). This automaticity is necessary to clearly and rapidly guide a person’s responses to their environment. Thus, where there is a discrepancy between a person’s verbal declarations and behavior, it is likely that their behavior may provide stronger evidence of their beliefs as it is these representations of their situation that are guiding their actions. It is also possible that some beliefs are formed before a person has acquired language, and so may be more difficult to articulate in linguistic terms (Young et al., 2003).

## CANDIDATE COGNITIVE PROCESSES

Belief formation is likely to involve a number of distinct cognitive processes. One promising avenues for explaining the nature of beliefs has come from the study of clinically disabling, strongly-held mono-delusional beliefs. Although some philosophers question whether delusions are pathological versions of belief (e.g., Currie, 2000), such accounts remain unconvincing because there is no empirical evidence to suggest that delusions are qualitatively different from the range of non-delusional beliefs (see Bayne and Pacherie, 2005; Bortolotti, 2009, 2013). In addition, the study of delusions has shown the potential to contribute much to the understanding of beliefs themselves. Over the last four decades, researchers have attempted to explain delusions in terms of breakdowns or dysfunctions to a variety of different putative normal cognitive processes. By proposing and examining the putative causes (i.e., pathology) behind delusions, it has been possible to come up with a number of candidate cognitive process (David and Halligan, 1996, 2000; Young, 2000; Halligan and David, 2001).

Cognitive theories of delusion formation generally fall into three camps:

• top–down processes that concentrate on reasoning, motivational influences, and cognitive biases
• those focusing on anomalous experiences that play a critical role in the belief process and
• a combination of both (see Bell et al., 2006b).

The top–down approaches tend to focus on non-bizarre (in particular, persecutory) delusions, whereas the second type often consider bizarre mono-delusions to illustrate their models (for a discussion of the distinction between non-bizarre and bizarre delusions, see Jaspers, 1963; Parnas, 2004; Bell et al., 2006c; Cermolacce et al., 2010). As can be seen from the model of persecutory delusion formation proposed by Freeman et al. (2002), this is often just a matter of emphasis (in reference to the particular delusion type in mind), with researchers acknowledging that both factors may actually play a role.

Across these approaches, several candidate cognitive processes have been proposed to contribute to or account for delusion formation (summarized in **Table 1**). Some of these have been developed with particular reference to certain types of delusions, whereas others have been hypothesized to play a role in the formation of all or most delusions. Given the varied nature of delusions, it seems likely that there are several routes to delusion formation, with these cognitive processes playing roles to differing degrees in different types of delusions.

**Table 1:** Some of the main cognitive factors proposed to contribute to delusion formation.

Proposed deficit/bias	Main account	Key proponents
Perceptual experience	Delusions are the result of normal reasoning applied to abnormal perceptual experiences	Reed (1972), Maher (1974, 1988)
Face processing	Capgras delusion stems from a covert affective face processing deficit (other misidentification delusions result from other face processing deficits)	Ellis and Young (1990, 1996), Ellis et al. (1997); Stone and Young (1997), Ellis and Lewis (2001)
Attribution processes	Persecutory delusions result from excessive attribution of negative events to other people in an attempt to protect self-esteem	Bentall et al. (1994, 2001)
Inferential reasoning	“Jumping to conclusions” reasoning style causes delusional beliefs to be formed from low levels of perceptual information despite pre-existing knowledge	Hemsley (1993), Garety and Hemsley (1994), Garety and Freeman (1999, 2013)
Belief evaluation	In response to anomalous experience or data, a deficit/bias in belief evaluation leads to the acceptance of an unlikely hypothesis as belief	Langdon and Coltheart (2000); Coltheart (2007, 2010), Coltheart et al. (2011)
Metacognitive beliefs	Delusions result from information that is accurately perceived but is misinterpreted due to faulty self and social knowledge	Morrison (2001)
Metarepresentation	Delusions of reference, misinterpretation and persecution may result from misinterpretation of another person’s behavior or intentions; delusions of control may result from losing the ability to identify self-generated thoughts and actions as one’s own	Frith (1992)
Cycle of preconscious perceptual processing	Preconscious expectancies, driven by existing beliefs, facilitate the interpretation of perceptual information, which in turn reinforces these beliefs	Fleminger (1992)
Interpretive frenzy and reference focus	Unconstrained, excessive inferences and ideas of reference due to left hemisphere overactivity lead to delusional content	Braun and Suffren (2011)
Disturbance in error-dependent updating of inferences	Inappropriate “prediction errors” – the mismatch between expectancy and experience – lead patients to attend to and infer relationships between unrelated events	Fletcher and Frith (2009), Corlett et al. (2010)
Effect of delusion on experience	A delusion restructures interpretation of sensory experience, such that the patient experiences the world as if the delusion were true, thereby reinforcing the delusion	Stone and Young (1997); Davies et al. (2001), Young (2008, 2010)
Reciprocal relationships between delusions and memory	A delusion encourages encoding and retrieval of memories that are consistent with the delusion’s content, which, in turn, reinforces the delusion.	Berna et al. (2014)

A number of accounts have focused on persecutory delusions (the belief that others are conspiring to cause one harm), a frequently reported type of delusion. Freeman et al. (2002), for example, outlined a number of factors that could cause this delusion within a biopsychosocial model. According to this account, some individuals are vulnerable to delusions due to a range of social, genetic, biological, and psychological factors, particularly in times of stress. As a result, these individuals may have anomalous experiences, such as hallucinations, intrusive thoughts, or actions that appear involuntary. Some individuals, in searching for an explanation for these anomalous experiences, can be influenced by cognitive biases, emotions, and pre-existing beliefs that emphasise the notion of threat. The jumping-to-conclusions bias, for instance, in which participants arrive at decisions using very little information may persuade individuals to quickly accept explanations that might otherwise be considered implausible. Anxiety, as well depression and anger, might lead to explanations based on threat. Pre-existing beliefs, particularly those that consider the world as hostile and the individual susceptible to threat, may also lead to persecutory explanations. As a consequence of these processes, explanations arising in a search for meaning and that are based on the idea of persecution may become accepted as belief (Freeman, 2007).

Other theorists have emphasised different deficits that may contribute to persecutory delusions. According to Frith (1992), deficits in meta-representation and theory of mind (the ability to infer and reason about mental states in others) can also lead to delusion formation. As a result of such deficits, patients may come to misinterpret the intentions and actions of others as hostile, leading to poor social outcomes. In contrast, Bentall et al. (1994, 2001) suggest that attributional biases, designed to compensate for low self-esteem, may also generate persecutory ideation. In particular, to avoid negative views of themselves reaching awareness, some individuals display excessive externalizing and personalizing biases – where they attribute negative events to the harmful intentions of other people, rather than to themselves or circumstances. While evidence for the deficits proposed by Frith and Bentall remains unclear (Garety and Freeman, 1999, 2013; Freeman, 2007), both deficits can be readily incorporated into Freeman and colleagues’ more general model of persecutory delusions.

Other accounts have focused on more bizarre delusions. These theories have likewise assumed that the content of delusions may arise from the person’s attempt to explain their unusual experiences (James, 1890; Reed, 1972; Maher, 1974, 1988). Ellis and Young (1990), for example, observed that the content of misidentification delusions could be explained in terms of various disruptions to normal face processing, including person identification. In the case of Capgras delusion (the belief that a familiar person has been replaced by an impostor), for example, damage to an autonomic response in face processing can lead some patients to lose their heightened arousal to familiar faces. As a result, patients encounter their loved ones without the normal heightened arousal they would expect to experience, which may lead to the idea that a familiar person has been replaced by a look-alike impostor (Ellis and Young, 1990, 1996; Stone and Young, 1997). In support of this account, a number of studies have found that patients with Capgras show reduced autonomic responses (indexed by skin conductance recordings) to photographs of familiar faces and similar low levels of autonomic response to familiar and unfamiliar faces (Ellis et al., 1997; Hirstein and Ramachandran, 1997; Brighetti et al., 2007).

While going somewhere toward accounting for the content of Capgras delusion, this bespoke face-processing account alone was insufficient and had difficulty explaining the maintenance of delusions and other types of delusion. It also had difficulty accounting for many patients with face-processing deficits who did not develop face-processing related delusions (Tranel et al., 1995). To account for such cases, Langdon and Coltheart (2000) proposed a generic **two-factor theory**. According to this account, two separate factors were considered responsible, working in combination to produce a delusion’s content and its subsequent maintenance. The first pathology factor involving the normal system (Factor 1) explains the delusion’s content and typically involves a neuropsychological anomaly affecting perceptual, emotional, or autonomic processing. In the case of Capgras, the person’s face does not elicit the usual autonomic response. The second pathology factor (Factor 2) helps explain the delusion’s maintenance and involves a deficit in a hypothetical normal belief evaluation and revision system. Patients who have both pathology Factors 1 and 2 are therefore likely to develop a delusion (McKay et al., 2005; Coltheart, 2007, 2010; Coltheart et al., 2011). While the two-factor theory is an important account of delusions, it does not fully explain why the delusional explanation for Factor 1 is chosen when a number of other alternate explanations are also possible. In addition, large numbers of people also claim to hold clinically similar unusual beliefs (Pechey and Halligan, 2011) and it is unlikely that all are due to neuropsychological damage (cf. Coltheart et al., 2011).

Another influential theory is the “prediction error” account of delusions (Fletcher and Frith, 2009; Corlett et al., 2010). According to this model, inappropriate “prediction errors” – the perceived inability of existing beliefs to account for sensory experience – can predispose patients to attend to and infer relationships between unrelated events. When viewed from the framework of a two-factor account, such aberrant prediction error may explain the content of the delusion. It may explain, for example, why particular stimuli become salient to the individual and the focus of delusional beliefs. Abnormal prediction errors may also provide an account for the maintenance of the delusion as well. According to Corlett et al. (2009), aberrant prediction errors could re-evoke the content of the delusion over time in a way that leads to reconsolidation of the delusion in the absence of any supporting evidence (Corlett et al., 2009). Thus, prediction error offers a parsimonious single-factor account of delusions. Many details of this model and the empirical evidence supporting it are still subject to discussions (Griffiths et al., 2014). Nevertheless, although proposing a single factor, the prediction error account appears to posit different mechanisms to account for how a delusion is formed and maintained. So it appears that the distinction of content and maintenance remains important, at least conceptually, to account for delusions.

## STAGES OF BELIEF FORMATION

**FIGURE 1:**
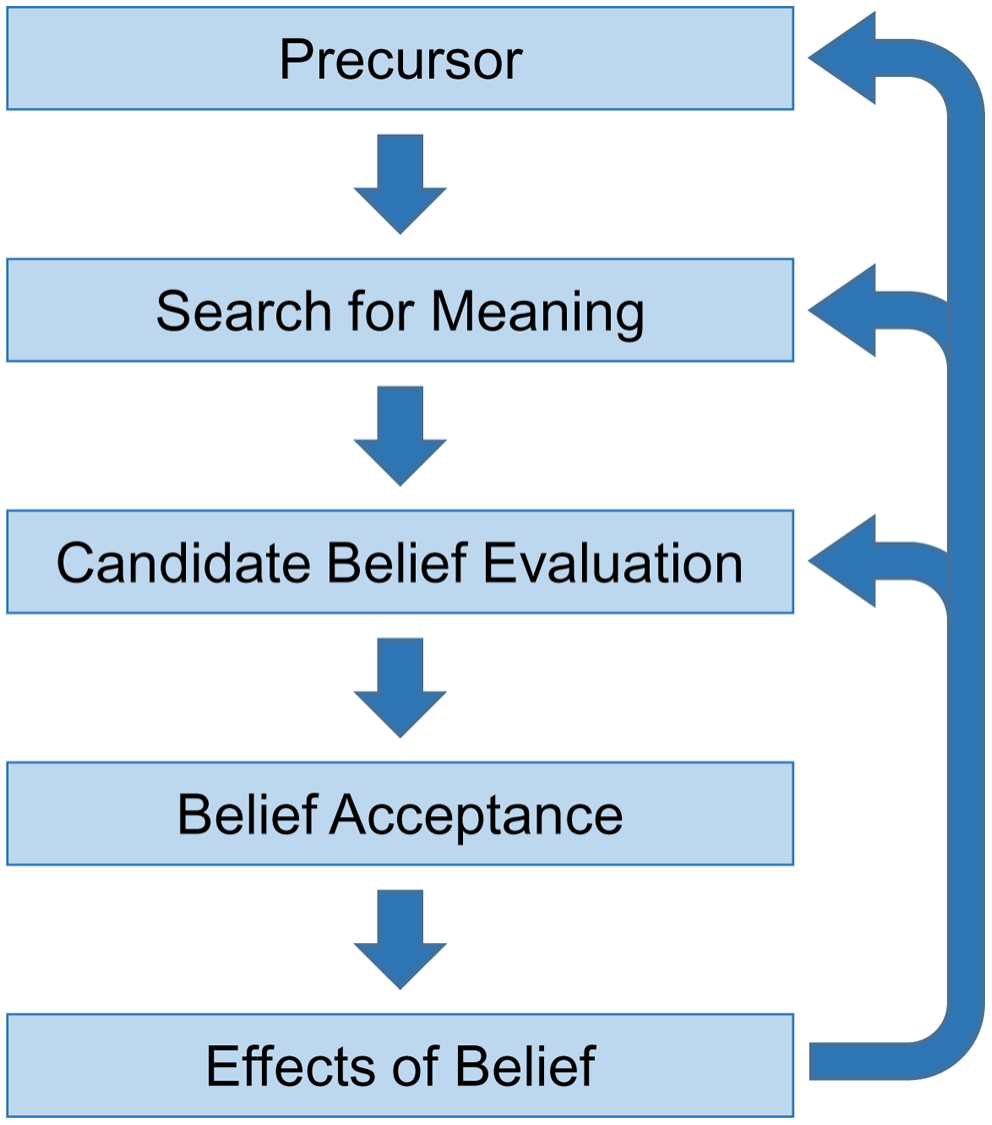
**A non-recursive five-stage account of belief formation**.

Based largely on evidence from clinical delusions and existing cognitive accounts of these pathologies, it is likely that a complete model of belief formation will involve a number of distinct stages. In this section, we provide a tentative five stage non-recursive account (see **Figure 1**). Given its dependency on delusion research, we also briefly note, where relevant, how these stages might inform understanding of clinical delusions. It should be emphasised, however, that subjects are most likely not aware of these stages as many of the cognitive process involved occur automatically and outside of conscious awareness. It also remains an empirical question as to whether all types of delusions can be understood within a framework of normal belief formation (see Jaspers, 1963; Parnas, 2004; Bortolotti, 2009, 2013; Cermolacce et al., 2010).

### I. PRECURSOR

The first critical stage is a precursor, which can be viewed as the trigger stage in belief formation. This distal trigger helps shape and determine the content of a yet-to-be generated new belief (a proto-belief). For many beliefs, the precursor may comprise a veridical or impaired perceptual input that subsequently initiates the subject’s search for meaning. This can occur when an unexpected perceptual input occurs or is unusual, so does not match with a person’s current expectations and existing web of beliefs (Fletcher and Frith, 2009; Corlett et al., 2010). Alternatively, the perceptual input may be emotionally salient or self-relevant in some way. In either case, the precursor triggers active monitoring processes to detect and explain the input. In the case of clinical delusions, anomalous experiences, such as those arising from a neuropsychological deficit to primary cognitive systems, have been regarded as a potent and consistent precursor (Langdon and Coltheart, 2000; Connors et al., 2015). It is also possible, however, that unusual (i.e., inexplicable) environmental events can lead to incomplete or inferentially incorrect explanations without any structural pathology.

Not all new beliefs, though, need to arise from perturbations of direct experience (Sperber, 2009; Sperber et al., 2010). Beliefs can stem from interaction with other people and media in our social environment, or from secondary informational sources, such as books, newspapers and television. In such cases, a precursor might be a verbal or written communication. As already noted, the ability to communicate beliefs serves important social functions, such as facilitating group cohesion and co-ordination. In the case of delusions, socially transmitted ideas could also precipitate delusional content without the need for an unusual experience or structural pathology. Delusional ideas in a person’s immediate environment, for example, can lead to shared delusions, whereby two or more people come to hold the same delusional belief (Hughes and Sims, 1997; Langdon, 2013). Alternatively, ideas from a person’s social and cultural environment can provide a precursor for a delusion. This is evident, for example, in delusions that incorporate specific technologies (e.g., Bell et al., 2005; McNally and Clancy, 2005) or involve themes that are specific to a historical period (e.g., Speak, 1990; Škodlar et al., 2008; Cannon and Kramer, 2011; Gold and Gold, 2012, 2014). In both cases, ready-formed social ideas may form the precursor to a belief without the need for further interpretation.

Beliefs can also arise from conscious or unconscious introspection. This includes, in particular, cognitive reappraisals of past events or perturbation to pre-existing beliefs. Sperber (1997), for instance, provides the example of asking someone whether there are any kangaroos on Jupiter. The answer to this question is unlikely to be already stored in memory as a belief (unless the person answering it has already heard the question before). However, on the basis of other pre-existing beliefs, a person may quickly derive an answer. In these cases, the precursor may be the pre-existing beliefs or the stimulus that instigated the search. As a result, pre-existing ideas or autobiographical past memories may provide the trigger for a new belief, providing they become salient in some way.

### II. SEARCH FOR MEANING

The second critical stage of the belief formation process is the search for meaning. This involves explaining or accounting for the experienced precursor and accommodating it within the existing web of beliefs. This search for meaning may draw upon pre-existing beliefs and other information relevant to the input, but is also guided by the constraint of avoiding cognitive inconsistency. This stage accounts for how the precursor acquires the specific meaning(s) it does and likely involves abductive reasoning – reasoning to the best explanation for the observed phenomena while accounting for pre-existing beliefs (Johnson-Laird, 2006; Coltheart et al., 2010). The outcome of this stage could result in several *proto-beliefs* or candidate proposals to account for the perturbation. This search for meaning to produce proto-beliefs is likely to be a mandatory and automatic process that complex cognitive systems are programmed to initiate and satisfy, particularly given the potentially destabilizing consequences of protracted uncertainty.

As any search for meaning is likely to be strongly constrained by pre-existing beliefs, certain explanations are more likely to be favored or competitive. Associations between the content of certain beliefs may also be more readily acquired than others. There is evidence, for example, that people more readily develop a phobia of snakes than for power sockets, which can be just as dangerous (Seligman, 1971). Likewise, there is evidence at a population level that the degree of belief co-endorsement between beliefs within thematic groupings is greater than random occurrence (Pechey and Halligan, 2012a). As a result of these constraints, the amount of inference required in a search for meaning may vary depending on the nature of the precursor. When an observation is unambiguous or appears highly consistent with pre-existing beliefs, it may simply be accepted as veridical without any attempt at further explanation. Alternatively, if the observation is more ambiguous, a greater amount of inference and cognitive effort may be required to generate an explanation (see Davies et al., 2001; Langdon and Bayne, 2010).

Given that any search for meaning will largely depend on pre-existing beliefs and knowledge, the outcome is likely to be highly personal and idiosyncratic. Overarching narratives that are implicit in subjects’ pre-existing beliefs may be particularly influential in determining the outcome of the search. In addition, subjects may adopt particular attributional styles – habitual tendencies to explain events in certain ways (Kelley and Michela, 1980) – whilst also relying on heuristics to save on cognitive effort (Kahneman et al., 1982; Gigerenzer and Gaissmaier, 2011; Kahneman, 2011). Subjects’ emotion and mood may also be relevant influences at this stage. Explanations may be selected because they are congruent with a prevailing emotion or dominant mood. Anxiety, for example, may foster explanations involving threat or danger, whereas happiness might prompt more benign explanations. Alternatively, explanations may be selected based on their affective consequences (Kunda, 1990; Gilovich, 1991; Helzer and Dunning, 2012). Explanations, for example, that offer certainty and comfort or maintain self-esteem and internal consistency are more likely to be selected over other explanations that do not provide these benefits, providing they are sufficiently plausible and can be rationalised. Motivation and emotion may constitute a particularly powerful determinant of evaluative beliefs (Kruglanski and Stroebe, 2005).

It remains unclear, however, the degree to which the hypotheses and proto-beliefs are scrutinised at this initial stage. Gilbert and colleagues have juxtaposed what they term Cartesian and Spinozan views of belief (Gilbert, 1991; Gilbert et al., 1993). According to a Cartesian view, the initial formation of a hypothesis – a proto-belief – requires further evaluation in a subsequent stage before the belief is adopted or accepted. In contrast, according to a Spinozan view, the initial formation of a hypothesis also entails the temporary adoption of that hypothesis; only once it is adopted as belief will it be further assessed to determine whether it will be maintained or rejected (see Davies and Egan, 2013). Gilbert and colleagues presented some evidence that the Spinozan account – namely the formation of the proto-belief involves some temporary acceptance of the proto-belief – is the more likely of the two. Nevertheless, while the accounts differ in terms of the degree to which the initial hypothesis is adopted before further scrutiny, both accounts agree that some form of further belief evaluation is likely to occur in belief formation (Gilbert, 1991; Gilbert et al., 1993).

In the case of delusions, the search for meaning also plays a critical role. As already noted, delusions can result from attempts to explain an anomalous experience or precursor (Maher, 1974; Coltheart et al., 2011). Importantly, however, these attempts are likely to be strongly influenced by a person’s pre-existing background, knowledge, and beliefs. Attributional style, heuristics, and cognitive biases that are present to varying degrees in the normal population may also lead subjects to favor certain explanations over others (Kihlstrom and Hoyt, 1988). In addition, the search for meaning could be constrained by selective impairment or injury. Cognitive deficits, for example, could lead patients to prioritise emotion-driven explanations or initial hypotheses on the basis of immediate experience. So while a precursor might strongly influence the content of a delusion, it cannot be said to fully determine it. The final content of the delusion arises only following the search for meaning to explain it.

The search for meaning thus helps to explain the observed variability within clinically presented delusions, as individuals may select and entertain different proto-beliefs to account for similar precursors. Individuals, for example, may conclude that their loved one has been replaced by a robot rather than an impostor (as in variants of Capgras), or that organisations are physically spying on them rather than reading their thoughts (as in variants of persecutory delusions). These proto-beliefs may also be influenced by the initial response of family, friends, and clinicians.

The search for meaning also helps to explain why not all individuals who experience neuropsychological anomalies develop delusions warranting clinical attention. Individuals may simply select a non-delusional explanation due to pre-existing beliefs or social input. When a delusional account is generated, it may be chosen because no alternative explanations are readily available, the delusional account is simply the most compelling (Freeman et al., 2004), or dysfunction in some or all stages of belief formation process have produced a sub-optimal search for meaning.

Delusions, however, may arise in the search for meaning stage without an anomalous precursor that bears an obvious resemblance to the final belief. Some perturbation or corruption of existing belief systems could lead to the interpretation of innocuous stimuli in the environment in a way that is considered to be delusional. Once an individual adopts an unusual belief or delusion, for example, it behoves them to interpret and re-interpret other aspects of their experience in line with the pre-existing belief. Pre-existing delusions therefore contribute to the sematic filter that is applied to attempts to explain anomalous data. This, in turn, perpetuates the original delusion and may potentially produce other related and supportive unusual beliefs.

In a similar way, ideas from a person’s social and cultural environment could influence the search for meaning and lead to delusional content without an obvious precursor. Individuals who trust or depend on people with delusions, for example, might come to adopt these delusions as explanations for events that occur in their own lives (Hughes and Sims, 1997; Langdon, 2013). Ideas derived from a person’s broader cultural environment can also lead to a delusion by providing a ready-made account of phenomena. As already noted, for example, there are a range of delusions that incorporate information from specific social and cultural contexts in their content (Hsia and Tsai, 1981; Speak, 1990; Chowdhury, 1996; Tateyama et al., 1998; Stompe et al., 1999; Bell et al., 2005; McNally and Clancy, 2005; Gold and Gold, 2012, 2014), as well as evidence of changing themes in delusions over time (Škodlar et al., 2008; Cannon and Kramer, 2011). In these cases, shared social and cultural ideas bias and shape the search for meaning to produce a pathological belief.

### III. CANDIDATE BELIEF EVALUATION

The third critical stage is the evaluation of the competing proto-beliefs in terms of their ability to account for the precursor and their consistency with pre-existing beliefs. Proto-beliefs need to be both observationally adequate (i.e., explain the precursor), yet also consistent with existing beliefs (Stone and Young, 1997; McKay, 2012). As a result, the belief evaluation process may vary across individuals with different beliefs and particular reasoning biases. For the most part, it is likely that there will be a predisposition toward conserving existing beliefs to avoid perturbations to internal consistency (Gilovich, 1991). Thus proto-beliefs that are more consistent with pre-existing beliefs are more likely to be accepted with less scrutiny or cognitive effort. In contrast, proto-beliefs that do not fit with pre-existing beliefs may be regarded as less competitive. In this case, people may resist the challenge of a proto-belief that is inconsistent with their existing beliefs – what they may consider to be anomalous – by subjecting it to particularly intense scrutiny, possibly even at a conscious level (Lord et al., 1979; Gilovich, 1991; Halligan et al., 1993).

Belief evaluation, even in the absence of frank pathology, has several limitations. People tend to adopt non-optimal hypothesis-testing strategies (Evans, 1989; Gilovich, 1991; Johnson-Laird, 2006; Nickerson, 2008). People, for example, tend to seek confirmatory information that supports their belief and be overly influenced by this information, but neglect information that is critical of their belief (Nickerson, 1998, 2008). People may also use inefficient strategies that waste effort on non-diagnostic data (Fischoff and Beyth-Marom, 1983; Evans, 1989; Johnson-Laird, 2006) or focus on heuristics (Kahneman et al., 1982; Gigerenzer and Gaissmaier, 2011; Kahneman, 2011; see also Gilovich et al., 2002). Indeed, the heuristic of anchoring and adjustment, which reflects the general tendency to rely on initial judgements and discount newly obtained information, means that knowledge received after the initial judgment may be distorted to fit the original hypothesis. In support of this, there is research suggesting that beliefs may persevere even when the initial evidence for the beliefs is discredited (Ross et al., 1975, 1977; Anderson et al., 1980). As a result of these biases, people can accept beliefs without sufficient evidence and also retain incorrect beliefs longer than would be case if they sought out diagnostic information. The collective impact of these tendencies is that people (i.e., their cognitive systems) are unlikely to seek information that contradicts their proto-belief, so long as the proto-belief is consistent with pre-existing beliefs or satisfies strong emotional drivers.

Emotion can also clearly bias belief evaluation. Mood states, for example, have been shown to influence the amount of effort individuals spend on processing information: individuals may be more highly motivated to scrutinise persuasive arguments (and therefore to be more influenced by the quality of these arguments) when in a negative rather than positive or neutral mood (Forgas, 2000). Similarly, individuals are more likely to recall memories that are congruent with their current emotions than memories that are incongruent (Mathews and MacLeod, 1994; Kihlstrom et al., 2000). The affective consequences of proto-beliefs may also bias evaluation, such that proto-beliefs which offer security and self-esteem may be less heavily scrutinised (Kunda, 1990; Gilovich, 1991; Helzer and Dunning, 2012). Emotion, mood, and motivational factors may thus influence both the level of scrutiny and the criteria used to evaluate proto-beliefs.

In the case of some delusions, particularly those involving bizarre and highly implausible content, a formal deficit in belief evaluation may be implicated in the uncritical acceptance of beliefs (Langdon and Coltheart, 2000; Turner and Coltheart, 2010). This deficit in belief evaluation could result in a tendency to be overly swayed by current experience, which provides evidence for a delusional explanation, and to downplay stored knowledge and past experience that would undermine the delusional explanation (Hemsley, 1993; McKay, 2012). As a result, ideas that would otherwise be rejected may instead be accepted as belief. Such a deficit, however, would not necessarily be required to explain all delusions, particularly those of more mundane content or that are consistent with other pre-existing beliefs.

The significant limitations of ordinary belief evaluation could also lead to the acceptance of unusual beliefs in the absence of pathology (Pechey and Halligan, 2011). The tendency to seek confirmatory evidence and be overly influenced by it, in particular, could lead to the acceptance or entertainment of unusual beliefs. Likewise, if an individual has strong pre-existing beliefs that are consistent with a delusional account, these beliefs might lead to acceptance of the delusional account without any additional deficit in belief evaluation. In this latter case, the new delusion would fit within the pre-existing web of beliefs, so would be accepted, while intact belief evaluation could serve to eliminate alternative, non-delusional accounts that are not consistent with the pre-existing web of beliefs.

### IV. ACCEPTING OR HOLDING THE BELIEF

Proto-beliefs that survive scrutiny become accepted beliefs, although as pointed out earlier, the subject may not necessarily be aware of this. Across other stages, subjects are unlikely to have access to many of the unconscious process involved, and may only become consciously aware of the belief when asked to reflect on it (see Halligan and Oakley, 2000). A person’s subsequent behavioral change, emotional response, evidential reflection, and reporting can provide evidence of the degree of conviction in such a belief. This conviction is likely to depend on the same two key criteria in belief evaluation, namely the extent to which the belief explains and predicts their experience of the world (i.e., its observational adequacy), and the degree to which the belief is congruent with other beliefs (i.e., conservation of pre-existing beliefs). Both criteria, however, may vary across time and across different contexts, so it is possible for some beliefs to vary in the conviction with which they are held. Delusions, likewise, can very over time and in different contexts (Bisiach et al., 1991; Sharp et al., 1996; Connors and Coltheart, 2011). Newly formed beliefs, however, that fit within a coherent, pre-existing web of other beliefs are likely to remain relatively stable over time.

### V. CONSEQUENTIAL EFFECTS OF HOLDING THE BELIEF

When a belief is accepted as true and held as such, it can have immediate effects on the overall cognitive system, though this may depend on the environmental opportunities for demonstrating the belief. New beliefs will contribute, depending on their immediate relevance, to configuring the person’s perception, memory, and action. As a result, the person will perceive the world in a way that is consistent with the new and congruent existing beliefs. There is, for example, considerable evidence that beliefs can act to bias the perception and interpretation of information so that it is consistent with the beliefs (e.g., Hastorf and Cantril, 1954; Lord et al., 1979; Jones and Russell, 1980; Vallone et al., 1985; Gilovich, 1991). Ambiguous information may thus be perceived in a way that fits preconceptions, and so lead to the elaboration and extension of the existing beliefs. By updating a subject’s web of beliefs, the new belief also influences future attempts to explain unusual events and may constrain what other proto-beliefs can be accepted.

Beliefs, whatever their neural or cognitive structure, may ultimately depend upon multi-distributed memories systems for their retention and accessibility. Beliefs, however, can also have a powerful and direct impact on memory. Once beliefs are formed, they promote the encoding and retrieval of memories that are consistent with the cognitive and emotional content of the beliefs (Conway, 2005; Berna et al., 2014). In addition, retrieved memories may be reinterpreted, or even distorted, to fit these beliefs (see Loftus, 2004; Schacter et al., 2011). Repeated retrieval may reinforce beliefs in other ways as well. Repeated retrieval of memories, for example, may lead to the gradual loss of their details, such that the memories become increasingly summarized in a more abstract form. These abstract representations, in turn, may eventually come to contribute to the content and elaboration of beliefs (Berna et al., 2014). As a result of these various processes, memories remain largely coherent with beliefs and serve to reinforce them. Independent of these effects, subjects may act on or publically endorse new beliefs. This commitment – or subjects’ memory of their relevant behavior – can, by itself, contribute to the belief’s maintenance (Festinger, 1962; Bem, 1967). For most beliefs, however, it is likely that subjects remain unaware of the belief and only experience the belief’s cognitive, behavioral, and emotional effects.

These processes are also likely to occur in delusions. In the same way as other beliefs, delusions may lead people to interpret ambiguous information in a way that is consistent with their belief. There is evidence, for example, that people with paranormal beliefs tend to misinterpret normal events as paranormal (Ayeroff and Abelson, 1976; Benassi et al., 1979; Jones and Russell, 1980; Singer and Benassi, 1981; Irwin, 2009). In so doing, delusions may thus configure other cognitive systems, such as those of attention, perception and memory, to experience the world in a way that is consistent with the delusion. In the case of Capgras delusion, for example, a person may not only believe that their loved one is an impostor, but come to *experience* their loved one as an impostor when interacting with them (Young, 2008, 2010). This may serve to reinforce and maintain the belief in the absence of any other supporting evidence. Likewise, delusions likely determine the encoding and retrieval of memories in ways that support and reinforce the delusional beliefs (Berna et al., 2014).

Furthermore, delusions likely influence a person’s search for meaning in future events. In this way, the delusion is further reinforced and extended as other events are incorporated as evidence for the delusion, even if the precursor itself is no longer present. The net result may be a systematised delusional framework, particularly if the delusion affects appraisals of ongoing events (Cummings, 1985; Roberts, 1992). Despite these widespread cognitive effects, however, it should be noted that not all individuals act on their delusions. Some individuals who develop a delusion without other impairments may recognise that other people would consider their belief to be highly implausible, and so choose not to express their belief or act on it. Indeed, for the most part, only individuals who publically express their delusions or act on them, such that it attracts the attention of family members or health services, may be recruited for formal research.

## FUTURE DIRECTIONS

Although admittedly underspecified and limited by the paucity of research, this non-recursive five stage approach to characterizing belief formation and acceptance has the merits of being relatively parsimonious in the preparation of a more comprehensive integration of findings from cognitive and neuropsychological studies. We suggest that a complete theory of belief will need to account for at least these five stages. We acknowledge, however, that there are a number of challenges to investigating belief and developing a more comprehensive theoretical model. A particularly significant challenge is bridging the gap between research on delusions and laboratory-based research with healthy participants. Here, the multidimensional nature of belief requires careful consideration when operationalizing belief for experimental research. Similar beliefs, for example, can arise from direct experience or from accepting the testimony of trusted others, which can make it difficult to isolate specific mechanisms. Likewise, some processes, such as social influence, can affect a number of different stages of belief, which can also make it difficult to isolate specific stages. In addition, beliefs, even those espousing the same content, can very considerably in other properties, such as the conviction with which they held, their degree of preoccupation, and their impact on behavior, which presents further practical challenges.

In future, existing methodologies from many areas of cognitive science may be readily adapted for investigating and characterizing the cognitive architecture of non-pathological belief formation, maintenance, and revision. Areas of research that involve cognitive representations of the external environment – such as social cognition, memory, attitudes, social influence, and top–down influences on attention and perception – are all likely to be influenced by existing belief in some capacity and so may be particularly relevant. In addition to delusions, other pathologies may also provide insight into belief. Anosognosia – the denial of impairment, such as physical incapacity after stroke – shares many similarities to delusion and may provide important insights into how contradictory evidence is processed and managed (Vocat et al., 2013). Obsessive-compulsive disorder – an anxiety disorder involving intrusive thoughts and compulsive behaviors – can, in some cases, involve a dissociation between belief (e.g., knowing that the door is locked or stove is turned off) and behavior (e.g., repeated checking and rituals; Szechtman and Woody, 2004). As such, it may be particularly relevant to understanding the effect of belief on action. Hallucinations may also be informative about belief if one compares patients with insight (and who do not believe in the external reality of their hallucinations) to those without (who believe in the external reality of their hallucinations). As in other areas of cognitive science, however, it is likely that specific paradigms will need to be developed to investigate the underlying processes and dimensions of belief.

Despite these challenges and the paucity of current research, belief is too important a topic to not be the subject of formal investigation in its own right. Beliefs provide the basis for interacting with the world and are intimately involved in co-ordinating many other cognitive processes. Beliefs are also central to many social processes and provide the basis for identity, social cohesion, and social conflict. Moreover, beliefs are critical to understanding many psychiatric and psychological pathologies that cause great suffering. Thus, in addition to possible clinical applications, greater insight into the cognitive processes of belief promises a better understanding of cognitive systems, social dynamics, and ourselves.

## Conflict of Interest Statement

The authors declare that the research was conducted in the absence of any commercial or financial relationships that could be construed as a potential conflict of interest.
